# Recurrent massive haemoperitoneum associated with ruptured corpus luteum in women with congenital afibrinogenemia; case report

**DOI:** 10.4274/tjod.04935

**Published:** 2014-12-15

**Authors:** Özhan Özdemir, Mustafa Erkan Sarı, Aslıhan Kurt, Ertuğrul Şen, Cemal Reşat Atalay

**Affiliations:** 1 Ankara Numune Education and Research Hospital, Clinic of Gynecology and Obstetrics, Ankara, Turkey

**Keywords:** Congenital afibrinogenemia, ruptured corpus luteum, hemoperitoneum

## Abstract

Massive hemoperitoneum secondary to ruptured corpus luteum is a rare but serious and life-threatening complication for women with congenital bleeding disorders and may lead to surgical interventions and even oophorectomy. Congenital afibrinogenemia is a rare inherited coagulation disorder. As it can be asymptomatic, its clinical manifestations vary from minimal tendency of bleeding to life-threatening bleedings. Intraabdominal bleeding due to ovulation is very rare in these patients and only a few cases of corpus luteum rupture and hemoperitoneum in afibrinogenemic patients have been described. We report on a 28-year-old woman with congenital afibrinogenemia with recurrent massive intraabdominal bleeding due to ovulation as the presenting clinical sign. The first episode was managed with fresh frozen plasma, cryoprecipitate and blood transfusions; exploratory laparotomy and excision of the ruptured follicle was performed at the second episode; the third episode was managed with fresh frozen plasma, cryoprecipitate and blood transfusions; exploratory laparotomy and right salphingooopherectomy was performed at the fourth episode; fifth episode was managed with fresh frozen plasma, cryoprecipitate and blood transfusions. Conservative management is crucial for patients with congenital bleeding disorders. These case demonstrate that preservation of ovarian function is possible with a conservative approach and recurrent episodes may be prevented by suppression of ovulation.

## INTRODUCTION

Congenital afibrinogenemia is a rare, autosomal recessively inherited bleeding disorder which has an approximate incidence of 1/100.000, and is generally seen in children born in consanguineous marriages^(1)^. Fibrinogen is a glycoprotein synthesized from hepatocytes that has an important role in clot formation by turning into fibrin with the effect of thrombin. Fibrin provides the aggregation of thrombocytes by binding to glycoprotein IIb/IIIa on the surface of activated thrombocytes^(2)^. Epistaxis, hemarthrosis, muscular hematoma, gastrointestinal bleeding and menorrhagia are the most common symptoms in patients with afibrinogenemia, whereas massive intraabdominal bleeding due to ruptured corpus luteum has only been reported in a few cases^(3,4,5,6,7)^. Even if this condition is rare in women with congenital bleeding disorders, it may be a life-threatening complication even requiring oophorectomy^(3)^. Herein, we report a patient with a five-time history of massive intraabdominal bleeding due to ruptured corpus luteum who had to be performed oophorectomy.

## CASE

A 28-year-old patient with a history of spontaneous abortion applied to our clinic with stomach ache, nausea and dizziness. The first clinical evaluation revealed hypotension (100/65 mmHg) and tachycardia (97 beats/minute). She had guarding and rebound tenderness in abdominal exam with cervical sensitivity in pelvic exam. Her menstrual cycles were regular, and her last menstrual date was 18 days ago. She was learned to have gotten a diagnosis of congenital afibrinogenemia in early infancy. She had a history of twice laparotomies due to ruptured ovarian cysts; the first one was right ovarian cystectomy, and the second one was right oophorectomy about 4 years ago due to not having obtained hemostasis (Table 1). She had been advised to use combined oral contraceptives following surgery however she quit using them about a month prior to her appliance to our hospital as she was planning to get pregnant.

The transvaginal ultrasonography revealed a 32x28 mm hemorrhagic cyst with internal echogenity in the left ovary and prevalent intraabdominal fluid consistent with hematoma filling the whole pelvis being the deepest in the douglas pouch with a 35-mm depth. We detected her hemoglobin 9.9 g/dl, thrombocytes 341.000/µl, fibrinogen 0, and her INR, prothrombin time, and activated partial thromboplastin time were excessively long that they could not be measured as there was no coagulation. Owing to the hemoglobin level decreasing to 7.0 g/dl in the control blood count after two hours along with tachycardia and symptomatic hypotension, we transfused her with 2 units of erythrocyte suspension (ES) and 6 units of fresh frozen plasma (FFP). During the time we closely followed the vital signs, we continued to replace with cryoprecipitate, FFP and ES until the hemostasis was achieved. We discharged the patient when she was hemodynamically stable and stopped bleeding, after an ultrasonographic control showing no hemoperitoneum.

## DISCUSSION

Normally, intraperitoneal bleeding during ovulation does not cause a significant problem other than pelvic pain however in patients with congenital bleeding disorders or who use anticoagulants, it may lead to life-threatening conditions. The clinical case in congenital fibrinogen disorders may vary according to the level of fibrinogen deficiency. In congenital afibrinogenemia, no measurable level of fibrinogen is present. The patients typically start to bleed in the neonatal period, and, as in our case, 85% of them are diagnosed following an umblical cord bleeding^(2)^. The severity of the bleeding and frequency of attacks vary among individuals. In afibrinogenemia, tendency to bleed vary widely even among patients with the same mutations, and a number of modified genes are accused of this^(8)^.

The most common clinical cases in congenital afibrinogenemia are umblical cord and superficial mucosal bleedings (particularly menorrhagia and epistaxis). Musculoskeletal system bleedings such as hemarthrosis may also be observed in approximately half of the patients. Less frequently, gastrointestinal, urinary and intracranial hemorrhages may be seen. Besides these spontaneous bleedings, hemorrhages following minor traumas or interventional procedures may also occur^(9)^.

On the other hand, hemoperitoneum following a follicule rupture during ovulation is a rare but severe condition. A number of cases with coagulation disorders that were performed laparotomy, wedge resection of the ovaries, or even oophorectomy owing to hemoperitoneum due to ovulation or ruptured corpus luteum were reported in the literature^(3)^. In our case, we had to perform oophorectomy as hemostasis could not be achieved. In patients with coagulation disorders, through management of the bleeding episodes diminishes the rate of unnecessary surgical procedures as well as helping to preserve fertility in these patients.

Spontaneous thrombotic complications were paradoxically reported in patients with afibrinogenemia. Normally, fibrin prevents thrombin production by decreasing the activation of prothrombin. However, as there is no fibrin in patients with afibrinogenemia, no antithrombin activity is present. As a result, prothrombin activation and thrombin production increase. Furthermore, free thrombin leads to the synthesis of a couple of growth factors from thrombocytes causing the proliferation of vascular smooth muscle cells and intimal hyperplasia. In association with all these anomalies, thrombotic complications may be observed in these patients^(10)^.

The main approach in the treatment of bleeding episodes in patients with afibrinogenemia is to appropriately replace blood elements with fibrinogen, cryoprecipitate and FFP. In the management of spontaneous hemorrhages, target fibrinogen level is >1 g/L until hemostasis is achieved. The first choice for replacement should be fibrinogen concentrate in patients with afibrinogenemia. It is advantageous because it is virally inactive, its infusion volume is lower, and is less allergenic compared to other transfusion products. Cryoprecipitate and FFP may be alternatively chosen in emergent cases when fibrinogen concentrate cannot be obtained^(11)^. In our case, we had to use FFP and cryoprecipitate for this same reason. Thrombotic complications such as deep venous thrombosis and pulmonary embolization were reported in patients with afibrinogenemia, and the risk is higher when cryoprecipitate is used as it also contains other coagulation factors. For this reason, some authors recommend low molecular weight heparin prophylaxis during replacement^(12)^.

The use of prophylactic fibrinogen concentrates is debatable as it has risks of thrombosis, viral infections, and anti-fibrinogen antibody formation, leading to the fact that prophylactic replacement should be individualized^(13)^. Oral contraceptives are effective in preventing hemoperitoneum due to follicule rupture however they also increase the risk of thrombosis^(6)^. Our case had had a history of combined oral contraceptive use until a month ago, and menstrual bleeding had been observed due to ovulation just after quitting them.

During pregnancy, replacement is recommended to decrease the risk of abortion, placental detachment and postpartum bleeding. As fibrinogen has a role in placental implementation, the risk of spontaneous abortion is increased in the first 6-8 weeks of pregnancy in patients with afibrinogenemia who are not being replaced. Even in patients who are being replaced, placental detachment risk was shown to be high^(13)^.

## CONCLUSION

Massive intraabdominal hemorrhage following ovulation is rare but may still be observed in patients with congenital bleeding disorders such as congenital afibrinogenemia. In the management of these hemorrhages, conservative approach plays an important role in order to preserve fertility. In these patients, suppression of ovulation by combined oral contraceptives is a recommendable approach in order to prevent repeating hemorrhages.

## Figures and Tables

**Table 1:**
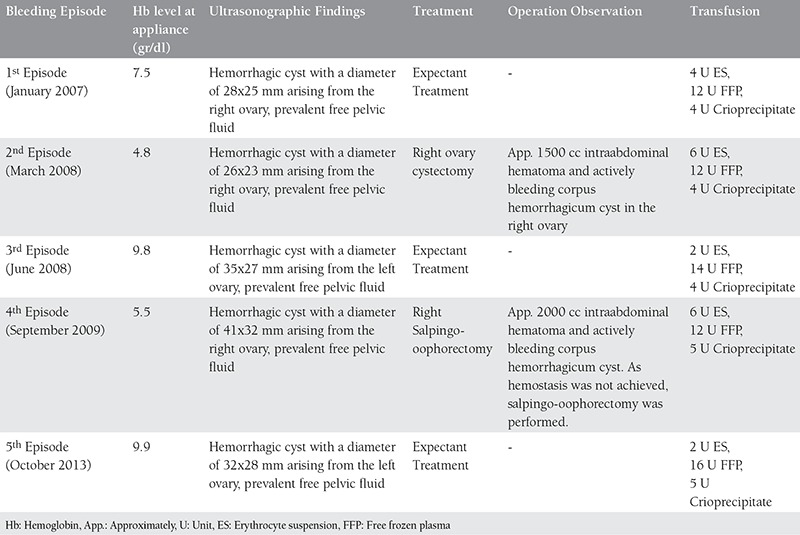
Chronologic bleeding episodes of the patient

## References

[1] Tziomalos K, Vakalopoulou S, Perifanis V, Garipidou V (2009). Treatment of congenital fibrinogen deficiency: overview and recent findings. Vasc Health Risk Manag.

[2] Acharya SS, Dimichele DM (2008). Rare inherited disorders of fibrinogen. Haemophilia.

[3] Cetinkaya SE, Pabuccu EG, Ozmen B, Dokmeci F (2011). Recurrent massive hemoperitoneum due to ovulation as a clinical sign in congenital afibrinogenemia. Acta Obstet Gynecol Scand.

[4] Schneider D, Bukovsky I, Kaufman S, Sadovsky G, Caspi E (1981). Severe ovarian hemorrhage in congenital afibrinogenemia. Acta Obstet Gynecol Scand.

[5] Bottini E, Pareti FI, Mari D, Mannucci PM, Muggiasca ML, Conti M (1991). Prevention of hemoperitoneum during ovulation by oral contraceptives in women with type III von Willebrand disease and afibrinogenemia. Case reports. Haematologica.

[6] Castaman G, Ruggeri M, Rodeghiero F (1995). Congenital afibrinogenemia: successful prevention of recurrent hemoperitoneum during ovulation by oral contraceptive. Am J Hematol.

[7] Koussi A, Economou M, Athanasiou-Metaxa M (2001). Intra-abdominal haemorrhage due to a ruptured corpus luteum cyst in a girl with congenital afibrinogenemia. Eur J Pediatr.

[8] Moerloose P, Neerman-Arbez M (2008). Treatment of congenital fibrinogen disorders. Expert Opin Biol Ther.

[9] Lak M, Keihani M, Elahi F, Peyvandi F, Mannucci PM (1999). Bleeding and thrombosis in 55 patients with inherited afibrinogenaemia. Br J Haematol.

[10] Oruc N, Tokat Y, Killi R, Tombuloglu M, Ilter T (2006). Budd-Chiari syndrome in an afibrinogenemic patient: a paradoxical complication. Dig Dis Sci.

[11] Bolton-Maggs PH, Perry DJ, Chalmers EA, Parapia LA, Wilde JT, Williams MD, et al (2004). The rare coagulation disorders - review with guidelines for management from the United Kingdom Haemophilia Centre Doctors’ Organisation. Haemophilia.

[12] Haberer JP, Obstler C, Samama CM, Darnige L, Szwebel TA, Meyer A, et al (2008). Postoperative deep venous thrombosis in a woman with congenital afibrinogenaemia treated with fibrinogen concentrates. Eur J Anaesthesiol.

[13] Kobayashi T, Kanayama N, Tokunaga N, Asahina T, Terao T (2000). Prenatal and peripartum management of congenital afibrinogenemia. Br J Haematol.

